# A synergistic blend of *Garcinia mangostana* fruit rind and *Cinnamomum tamala* leaf extracts enhances myogenic differentiation and mitochondrial biogenesis *in vitro* and muscle growth and strength in mice

**DOI:** 10.29219/fnr.v67.9750

**Published:** 2023-10-30

**Authors:** Swaraj Sinha, Krishnaraju Venkata Alluri, Venkateswarlu Somepalli, Trimurtulu Golakoti, Krishanu Sengupta

**Affiliations:** 1 Department of Cell and Molecular Biology, Laila Nutraceuticals R&D Center, Vijayawada, Andhra Pradesh, India; 2 Department of Pharmacology and Clinical Research, Laila Nutraceuticals R&D Center, Vijayawada, Andhra Pradesh, India; 3 Department of Phytochemistry, Laila Nutraceuticals R&D Center, Vijayawada, Andhra Pradesh, India

**Keywords:** ergogenic phytonutrients, mammalian target of rapamycin (mTOR), mitochondrial biogenesis, muscle growth and strength, nitric oxide, broad-spectrum safety

## Abstract

**Background:**

A proprietary combination of *Garcinia mangostana* fruit rind and *Cinnamomum tamala* leaf extracts (LI80020F4, CinDura^®^) improved the physical performance and muscle strength of resistance-trained adult males.

**Objective:**

This study assessed the underlying mechanisms of the ergogenic potential of LI80020F4 in *in vitro* and *in vivo* models.

**Methods:**

The individual extracts and their combination (LI80020F4) were assessed for nitrite production in EAhy926 human endothelial cells. Subsequent experiments evaluated the effect of LI80020F4 in myotube formation in C2C12 mouse myoblasts, expression of mammalian target of rapamycin (mTOR) signaling proteins, myogenic factors, and mitochondrial functions in L6 rat myoblasts.

Moreover, adult male ICR mice were randomly assigned (*n* = 15) into vehicle control (G1), exercise alone (G2), oxymetholone-16 mg/kg body weight (bw) (G3), and 75 (G4)-, 150 (G5)-, or 300 (G6) mg/kg bw of LI80020F4, orally gavaged for 28 days. G1 and G2 mice received 0.5% carboxymethylcellulose sodium. Following completion, muscle strength and physical performance were assessed on forelimb grip strength and forced swimming test (FST), respectively. Gastrocnemius (GA), tibialis anterior (TA) muscle weights, muscle fiber cross-sectional area (CSA), levels of muscle, and serum protein markers were also determined.

**Results:**

LI80020F4 increased nitrite production in EAhy926 cells in a dose-dependent manner. LI80020F4 induced C2C12 myotube formation, increased mitochondrial biogenesis, upregulated the expressions of activated mTOR and other mitochondria and myogenic proteins, and mitigated H_2_O_2_-induced mitochondrial membrane depolarization in the myoblast cells. In the animal study, 75, 150, and 300 mg/kg bw LI80020F4 doses significantly (*P* < 0.05) increased the animals’ forelimb grip strength. Mid- and high-dose groups showed increased swimming time, increased muscle weight, CSA, muscle growth-related, and mitochondrial protein expressions in the GA muscles.

**Conclusion:**

LI80020F4 increases nitric oxide production in the endothelial cells, mitochondrial biogenesis and function, upregulates skeletal muscle growth-related protein expressions and reduces oxidative stress; together, it explains the basis of the ergogenic potential of LI80020F4.

## Popular scientific summary

- LI80020F4 is a proprietary, synergistic composition of *G. mangostana* fruit rind and *C. tamala* leaf extracts that increase eNOS-dependent NO synthesis.
- LI80020F4 induces mitochondrial biogenesis, myogenic differentiation, and mitochondrial function in cultured cells.
- LI80020F4 shows ergogenic effects in experimental mice by increasing muscle mass, strength, and endurance.
- LI80020F4 provides antioxidant protection to the muscles mitigating exercise-induced muscle damage.

Over the past few decades, plant-derived nutraceuticals have become increasingly popular as dietary supplements consumed by sports enthusiasts and the general population to enhance muscle growth and function. Increased muscle mass and healthier functionality are critical for improved athletic or sports performance and general well-being (1–4). In the majority, botanical supplements modulate anabolic activities through select metabolic pathways or increase adaptogenic actions by enhancing the body’s resistance to exercise-induced stress or as stimulants that modulate the neuro-endocrine factors to increase alertness, stamina, and motivation, etc. (2, 5). However, a sustained high level of oxygen and nutrients supply to the working muscles through dilated blood vessels is critical for enhanced muscle adaptation and delayed fatigue, thus resulting in prolonged and improved exercise performance (6).

Nitric oxide (NO) is a potent vasodilator that induces various physiological activities, including vasodilation, increased blood flow, and mitochondrial respiration (7). In endothelial cells, endogenous NO is produced from the substrate L-arginine in the nitric oxide synthase (eNOS)-dependent pathway. NO induces vascular smooth muscle relaxation via increasing cyclic guanosine monophosphate (cGMP) levels utilizing guanylate cyclase (GC) activation, resulting in vasodilation (8, 9). NO-induced enhanced blood flow increases muscle growth and performance, improving strength training adaptations (10). Mitochondria are highly abundant in skeletal muscles and play a vital role in muscle function by regulating energy metabolism (11). Several studies reported a plausible link between eNOS-dependent NO production and improved mitochondrial efficiency through PGC1α upregulation and subsequent increases in aerobic metabolism and skeletal muscle performance (12–14). Enhanced mitochondrial number and function are essential for increased muscle adaptations to endurance and strength training (15).

Previously, in a proof-of-concept clinical study, LI80020F4 demonstrated significant ergogenic benefits in resistance-trained males. Consecutive 42 days of LI80020F4 supplementation increased the participants’ muscle growth, strength, and performance endurance (16). LI80020F4 is a proprietary composition containing aqueous-ethanol extracts of *Garcinia mangostana* fruit rind and *Cinnamomum tamala* leaf, combined at 1:2 ratio, and this composition is standardized to contain at least 3.5% α-mangostin and 0.1% rutin (16). In traditional medicine, *G. mangostana* fruit rind has been used to treat skin infections, abdominal pain, dysentery, and gastrointestinal and urinary tract infections (17). Earlier investigations reported that α-mangostin, a major xanthone, contributed to the anti-inflammatory, analgesic, antioxidant, and anti-lipogenic activities of *G. mangostana* fruit rind extracts (18). In Ayurveda, the medicinal properties of *C. tamala* leaf and bark have been described in treating rheumatism, cardiac disorders, colic, diarrhea, nausea, and vomiting (19). *C. tamala* leaves are rich in α-pinene, camphene, myrcene, limonene, p-cymene, and other phenolic compounds. *C. tamala* leaf extracts are anti-inflammatory, antioxidant, anti-hypercholesterolemic, anti-diarrheal, anti-microbial, anti-diabetic, and hepatoprotective (20, 21).

The present study describes LI80020F4 as a synergistic formulation of *G. mangostana* fruit rind and *C. tamala* leaf extracts that increase NO synthesis by eNOS activation. We evaluated muscle strength and endurance in a mouse model and also assessed critical markers of muscle protein synthesis and mitochondrial biogenesis *in vitro* and *in vivo*. Together, these observations provide the molecular basis of the ergogenic potential of this botanical composition.

## Materials and methods

### Test items

LI80020F4 (CinDura^®^) is a proprietary, standardized botanical formulation manufactured in a Good Manufacturing Practice (GMP) facility of Laila Nutraceuticals, Vijayawada, Andhra Pradesh, India. This unique phytoceutical comprises seven parts of a blend of aqueous-ethanol extracts of *Garcinia mangostana* fruit rind (GM) and *Cinnamomum tamala* leaf (CT) at a 1:2 ratio and three parts of excipients containing a mixture of microcrystalline cellulose and syloid. The extraction process of the individual dried plant materials and the standardization method of the finished product (LI80020F4) were described earlier (16). LI80020F4 is standardized to contain at least 3.5% α-mangostin and 0.1% rutin by the method of High-performance liquid chromatography (HPLC) (16).

### Reagents and chemicals

2, 3-Diaminonaphthalene dye (DAN), dimethyl sulfoxide (DMSO), phorbol 12- myristate 13-acetate (PMA), N,N–dimethyl-9,9 biacridinium dinitrate (Lucigenin), aprotinin, phenylmethylsulfonyl fluoride (PMSF), leupeptin, and pepstatin were purchased from Sigma Chemicals, St. Louis, MO. MitoBiogenesis™ In-Cell enzyme-linked immunosorbent assay (ELISA). kit, serum superoxide dismutase (SOD), and malondialdehyde (MDA) assay kits were procured from Abcam (Cambridge, UK). The antibodies to phospho-Ser2448 mammalian target of rapamycin (mTOR), phospho-AKT (Thr308), phospho-lactate dehydrogenase A (LDHA) (Tyr10), and their wild-type proteins, and succinate dehydrogenase complex subunit A (SDHA) were purchased from Cell Signaling Technology, Danvers, MA. Growth differentiation factor 8 (GDF-8, Myostatin), insulin-like growth factor 1 (IGF-1) ELISA kits, and MURF-1 antibody were purchased from R&D Systems Minneapolis, MN. ATP synthase and peroxisome proliferator-activated receptor-gamma coactivator (PGC)-1 alpha antibodies were purchased from Thermo Scientific, Rockford, IL, and Merck-Millipore, Billerica, MA, respectively. Atrogin-1 and sirtuin-1 (SIRT-1) antibodies were procured from Biovision, Milpitas, CA, and Santacruz Biotechnology, Dallas, TX, respectively. Oxymetholone was purchased from TCI Chemicals (India) Pvt. Ltd, Chennai, India. All other reagents used in this study were of analytical grade and procured from Sigma Chemicals, St. Louis, MO.

### In vitro studies

#### Cell culture and treatment

EAhy926 endothelial cells (CRL-2922), L6 (CRL1458), and C2C12 (CRL-1772) cells were obtained from American Type Culture Collection (ATCC, Manassas, VA) and cultured in a growth medium comprising DMEM supplemented with 10% fetal bovine serum (FBS; Gibco, Waltham, MA), 1% penicillin-streptomycin (Pen-Strep), 1 mM sodium pyruvate, and 4.5 g/L D-glucose. In all cell culture experiments, the desired number of cells suspended in growth media was equally distributed in each well and grown until 70–80% confluence before starting the treatments.

The primary stock solutions of the test samples GM, CT, or LI80020F4 were prepared in DMSO and stored in aliquots at −20°C. In all cell culture experiments, the test sample stock solutions were diluted to the desired final concentrations keeping the final DMSO concentrations consistent. The vehicle control (VC) culture wells received only the incubation medium containing the matched DMSO concentration (0.2%, v/v).

For immunoblot assay, the cells were serum starved for 16 h. Then, the cells were incubated for different periods in the presence or absence of 100 ng/ml LI80020F4. The cells incubated with 0.2% DMSO served as VC.

#### Nitrite assay

An equal number of EAhy926 cells suspended in the growth medium was seeded in each well of a 48-well cell culture plate (Corning Inc., NY) and incubated at 37°C for 24 h in a CO_2_ incubator. The culture wells were washed using a serum-free medium, and the cells were treated with or without different concentrations of GM, CT, or LI80020F4 in a serum-free medium containing 10 mM L-arginine for 24 h. Each treatment was carried out in triplicates. Cell-free culture supernatants were collected, and nitrite content was determined.

One hundred microliters of culture supernatant were placed in each well of a black 96-well microtiter plate (Corning Inc., NY). Twenty microliters of freshly prepared DAN reagent (30 µg/ml) were added to each well and incubated for 15 min at room temperature. The reaction was stopped by adding 50 µL of 2.8 N NaOH. Fluorescence was measured at excitation/emission: 360/430 nm using a fluorescence microplate reader (Spectramax M5e, Molecular Devices, Sunnyvale, CA).

#### Mitochondrial biogenesis assay

An equal number of L6 cells suspended in the growth medium was seeded on each well of a poly-L-lysine coated 96-well plate (Corning Inc. Corning, NY) and incubated overnight at 37°C in a CO_2_ incubator. The cells were treated with different concentrations of LI80020F4 for 72 h, with a repeat treatment every 24 h in a fresh growth medium. The VC culture wells received 0.2% DMSO (v/v). Mitochondrial biogenesis was determined using an in-cell ELISA kit (Abcam, Cambridge, UK), following the assay protocol provided by the manufacturer. Briefly, the treated cells were fixed with 4% paraformaldehyde and probed with primary antibodies against COX-1 (cytochrome c oxidase subunit I) and SDHA. The PBS-washed cells were reacted with alkaline phosphatase (AP) and horseradish peroxidase (HRP)-labeled secondary antibodies (Jackson Immuno Research, West Grove, PA) for the detection of SDHA and COX-1 expressions at 405 and 600 nm, respectively, in a multi-mode plate reader (Spectramax M2e, Molecular Devices, Sunnyvale, CA). The ratio between COX-I and SDHA expressions was calculated, and the percent (%) increase of mitochondrial biogenesis was determined in the treated cells over the VC cells.

#### Mitochondrial membrane potential assay

Mitochondrial trans-membrane potential (∆ψ_m_) of the LI80020F4-treated cells was measured using JC-1 dye (5, 5’, 6, 6’-tetrachloro-1, 1’, 3, 3’-tetraethylbenzimidazole-carbocyanide iodine) incorporation method (22). An equal number (1 × 10^6^) of L6 cells harvested from the log-phase cultures was taken into each sterile culture tube. The cells were treated with LI80020F4 at desired concentrations and incubated for 30 min in a CO_2_ incubator at 37°C. The 1X PBS-washed cells were then incubated with 5 mM H_2_O_2_, except for the VC (0.2% DMSO) and JC-1 control cells for 15 min in a CO_2_ incubator at 37°C. Except for the VC cells, the treated cells were further incubated with JC-1 (5 µg/mL in PBS) for 30 min in the dark at room temperature. The washed cells were re-suspended in 1X PBS and analyzed using a flow cytometer (BD FACS Verse, Franklin Lakes, NJ, USA).

#### Myotube assay

An equal number of C2C12 cells suspended in DMEM supplemented with 10% (v/v) FBS and 1% pen-strep solution was seeded in each well of a six-well cell culture plate containing a glass coverslip. The following day, C2C12 myoblasts were incubated in DMEM supplemented with 2% horse serum (Gibco, Waltham, MA), in the presence or absence of LI80020F4, at desired concentrations, over 6 consecutive days. The treatment was repeated on every alternate day.

Following treatment, the C2C12 myotubes on the coverslips were fixed with 4% paraformaldehyde and permeabilized with Triton X-100. The myotubes were incubated with myosin heavy chain (Santa Cruz Inc, Dallas, TX) antibody overnight at 4°C, followed by Alexa Fluor^®^ 488 AffiniPure Goat Anti-Rabbit IgG (H+L) (Jackson Immunoresearch, West Grove, PA) for 2 h. Finally, the coverslips were mounted on microscope slides and examined under phase contrast objectives. Fluorescent images were acquired (excitation/emission: 493/519 nm) using an Axio Observer Z1 microscope (Carl Zeiss, Oberkochen, Germany). The myotube diameters were measured using Fiji software (Image J version 1.2; WS Rasband, National Institute of Health, Bethesda, MD).

#### Western Blot assay

The treated cells were washed twice with 1X PBS, and the cells were lysed in a cell lysis buffer (10 mM Tris-HCl, pH 7.4, 150 mM NaCl, 1 mM EDTA, 1 mM PMSF, 10 μg/ml aprotinin, 10 μg/ml leupeptin, and 1% Triton X-100, 1 mM NaF, 1 mM Na_3_VO_4_, 0.5% (w/v) sodium deoxycholate, and 1 µM pepstatin). The cell lysates were clarified at 14,000 g for 10 min at 4°C. The mouse GA muscle samples were macerated in liquid N_2_ and homogenized in the cell lysis buffer. The tissue homogenate was centrifuged at 18,000 g for 20 min at 4°C. The protein concentration of the cell or tissue lysates was measured using a BCA protein assay kit (Merck-Millipore, Billerica, MA).

The western blot assays were performed following the methodology described earlier with some modifications (23). Briefly, an equal amount of cell or tissue lysate proteins was resolved using 7.5 or 10% sodium dodecyl sulfate-polyacrylamide gel electrophoresis (SDS-PAGE). The proteins were transferred onto nitrocellulose membrane, blocked with Superblock (Thermo Scientific, Rockford, IL) and reacted with the primary antibodies for 16 h at 4°C. Then the washed membranes were incubated with either HRP-conjugated goat anti-rabbit IgG (H + L) or goat anti-mouse IgG (H + L) (Jackson Immuno Research, West Grove, PA), and the antigen-antibody reactions were developed using a chemiluminescent substrate (Super Signal West Pico PLUS, Thermo Scientific, Waltham, MA). The protein band images were captured in a Chemi Doc XRS+ Imager (Bio-Rad, Hercules, CA). The antibody-stripped membranes were re-probed with β-actin or GAPDH antibody (Sigma Chemicals, St. Louis, MO) and developed to ensure equal protein loading. The protein bands were analyzed using molecular imaging software v.5.0.2.30 (Carestream Health, Rochester, NY).

### In vivo study

#### Experimental animals and study design

Adult male ICR mice (age: 7–8 weeks; body weight: 28–39 g) were purchased from Hylasco Biotechnology (India) Pvt. Ltd., Hyderabad, India. All animals were maintained under standard environmental conditions at 22 ± 3°C and 30–70% relative humidity with 12 h light/ dark cycle with free access to standard rodent pellet diet (Krishna Valley Agrotech Limited, Pune, India) and RO (Reverse Osmosis) water *ad libitum.* The experimental protocol was approved (Approval no: LI/IAEC/LI211013) by the Institutional Animal Ethical Committee (IAEC) of Laila Impex R&D Center, Vijayawada, India. The handling and care of the experimental animals were according to the guidelines of the Committee for the Purpose of Control and Supervision of Experiments on Animals (CPCSEA).

Following a 7-day acclimatization period, animals were randomly assigned into one of the six groups (*n* = 15): VC (G1), exercise alone (G2), oxymetholone- 16 mg/kg bw (G3; OXY), LI80020F4-75 mg/kg bw (G4), LI80020F4-150 mg/kg bw (G5), and LI80020F4-300 mg/kg bw (G6). The animals received 0.5% *w/v* carboxymethylcellulose sodium (CMC-Na) (G1 and G2), OXY (G3) or different doses of LI80020F4 (G4, G5, and G6) through oral gavage daily over 28 consecutive days. Except for G1 mice, the remaining animals received swimming training in a pool (water temperature 36 ± 2°C), without load, 3 alternate days (10 min each) per week. The forelimb grip strength and forced swimming test (FST) parameters were measured on days 27 and 28, respectively. The body weights were recorded every week. On day 29, the overnight fasting blood samples were collected, and following CO_2_ euthanasia, the gastrocnemius (GA) and tibialis anterior (TA) muscles were excised, weighed, and stored in aliquots at −70°C until further analyses. The muscle tissues were weighed using an analytical balance (Model# CP224S; Sartorius, Göttingen, Germany).

#### Forelimb grip strength measurement

The forelimb grip strength was evaluated using a grip strength meter (Laboratory Enterprises, Nashik, India), as described earlier (16). Briefly, all the mice were allowed to hold the grasping bar and were pulled back gently in a horizontal plane by their tails with gradually increasing force till the pulling force overcame the grip strength of the animal. The force applied at the moment the mouse leaves its clutch on the grasping bar while pulling by the tail was recorded as grip strength (in pound force) and was normalized using their body weight (pound-force/kg) and expressed in the SI unit (N/kg).

#### Weight-loaded FST

The FST was conducted in a cylindrical acrylic tank (20 cm diameter × 45 cm height) filled with warm water (36 ± 2°C) up to a depth of 30 cm, as described earlier (16). Mice swam in the water for 5 min with a load of 5% of their body weight on their tail. The parameters such as resting time (s), swimming time (s), and distance traveled (cm) were recorded and analyzed using the SMART video tracking system (PanLab S.L.U, Place, Barcelona/Spain).

#### Serum biochemical parameters

On day 29, the blood samples were collected through retro-orbital puncture under mild isoflurane anesthesia. The serum samples were tested for creatine kinase (CK), lactate dehydrogenase (LDH), blood urea nitrogen (BUN), aspartate aminotransferase (AST), and alanine transaminase (ALT) using an automated analyzer (ILab Aries^®^, Milano, Italy).

#### Muscle fiber cross-sectional area

At room temperature, GA and TA muscle tissue samples were fixed in 10% neutral buffered formalin for 24 h. The fixed tissues were processed in a series of ethanol grades and embedded in paraffin blocks. Five micrometers (µm) transverse sections of the paraffin-embedded tissues were mounted on clean glass slides and stained with Picro Sirius red. The stained tissue sections were examined under a microscope at 20X objective (Axio Vision observer Z1, Carl Zeiss GmbH, Jena, Germany). The bright field images were captured using a charge-coupled device (CCD) camera (ProgRes C5, Genoptik, Jena, Germany). The fiber cross-sectional area (CSA) was measured using Axiovision 4.8 software (Carl Zeiss GmbH, Jena, Germany), as described earlier (16). From each tissue section, 25 fibers were randomly selected to calculate the mean CSA.

#### Enzyme immunoassay

The GA muscle tissue lysates were analyzed for SOD, MDA, inorganic phosphorous (Pi) (Abcam, Waltham, MA), GDF-8 (Myostatin), and insulin-like growth factor-1 (IGF-1) (R&D Systems, Minneapolis, MN) using enzyme immunoassay (EIA) kits. The assays were performed following the manufacturer’s protocols. The assay sensitivities of the SOD, MDA, Pi, GDF-8 (Myostatin), and IGF-1 kits were 0.1 U/ml, 0.1 nmol, 1 µM, 5.32 pg/ml, and 8.40 pg/ml, respectively.

### Statistical analysis

A group size of 15 was chosen with an anticipated effect size (d) of ‘1.1’ and standard deviation (SD) of ‘2’ with a 90% power, 95% confidence interval (CI), and 10% attrition rate (*n* = 15). Fifteen animals per group were selected to represent an ‘E’ value of 84. Randomly selecting six animals’ samples for any parameter will give us an E value of 30. E value above 10 should be considered adequate for animal studies (24). The data were analyzed for normality using the Shapiro-Wilk test and expressed as mean ± SD. The comparative analyses among the different groups were performed by one-way and two-way analysis of variance (ANOVA) followed by Bonferroni post hoc test and Dunnett’s post hoc tests, respectively, using Graph Pad Prism software v5.01 (GraphPad Software Inc., San Diego, CA). A *P*-value < 0.05 was considered statistically significant.

## Results

### In vitro studies

#### LI80020F4 synergistically increased Nitrite (NO) production in EAhy926 cells

**Fig. 1:**
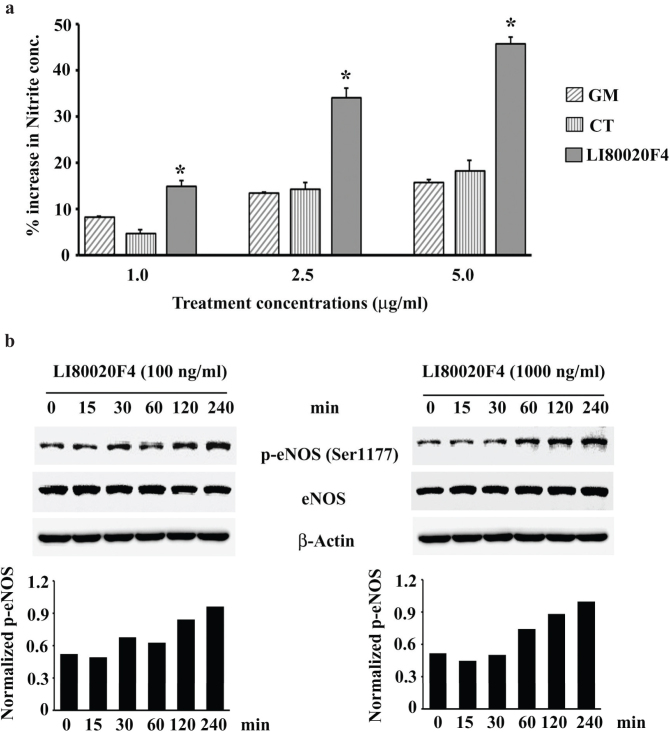
LI80020F4 increases nitrite production and phosphorylates eNOS in EAhy926 human endothelial cells. (a) Each bar represents the mean ± SD (*n* = 3) of percent increases in nitrite production in GM, CT, or LI80020F4 treated EAhy926 endothelial cell cultures at different concentrations, as indicated, compared to the vehicle control. (b) Representative western blot images show modulations of phospho-eNOS (Ser1177) protein expressions in 100 and 1,000 ng/mL of LI80020F4-treated EAhy926 cells at different time points, as indicated. The β-actin expression ensures equal protein loading. Bar graphs present normalized p-eNOS protein expressions (vs. total eNOS) in 100 and 1,000 ng/ml LI80020F4-treated cells at different time points, as indicated. GM and CT represent *Garcinia mangostana* fruit rind and *Cinnamomum tamala* leaf extracts. * indicates significance (*P* < 0.05) versus GM or CT.

Figure 1a presents increased nitrite concentrations in the GM (*G. mangostana* fruit rind extract), CT (*C. tamala* leaf extract), and LI80020F4-treated EAhy926 cell culture supernatants at the indicated concentrations. LI80020F4 treatment shows dose-dependent increases (14.85, 34.76, and 45.81% vs. VC) in nitrite levels (Fig. 1a). LI80020F4 treatments at 2.5 and 5 µg/mL increased nitrite productions by 2.56-fold (*P* = 0.0316; vs. GM) and 2.9-fold (*P* = 0.0056; vs. GM); 2.39-fold (*P* = 0.0088; vs. CT) and 2.53-fold (*P* = 0.0217; vs. CT), respectively (Fig. 1a). At 1 µg/ml, LI80020F4 treatment shows 1.84-fold (vs. GM; *P* = 0.0101) and 3.26-fold (vs. CT, *P* = 0.0120) increases in nitrite levels. Furthermore, representative immunoblot images show time-dependent increases in phospho-eNOS (Ser1177) protein expression in LI80020F4-treated endothelial cells. The bar diagrams present normalized p-eNOS protein expressions (vs. total eNOS) in a time-dependent manner (Fig. 1b).

#### LI80020F4 increases C2C12 myotube thickness, phosphorylates mTOR, and upregulates skeletal muscle-specific protein expressions

**Fig. 2:**
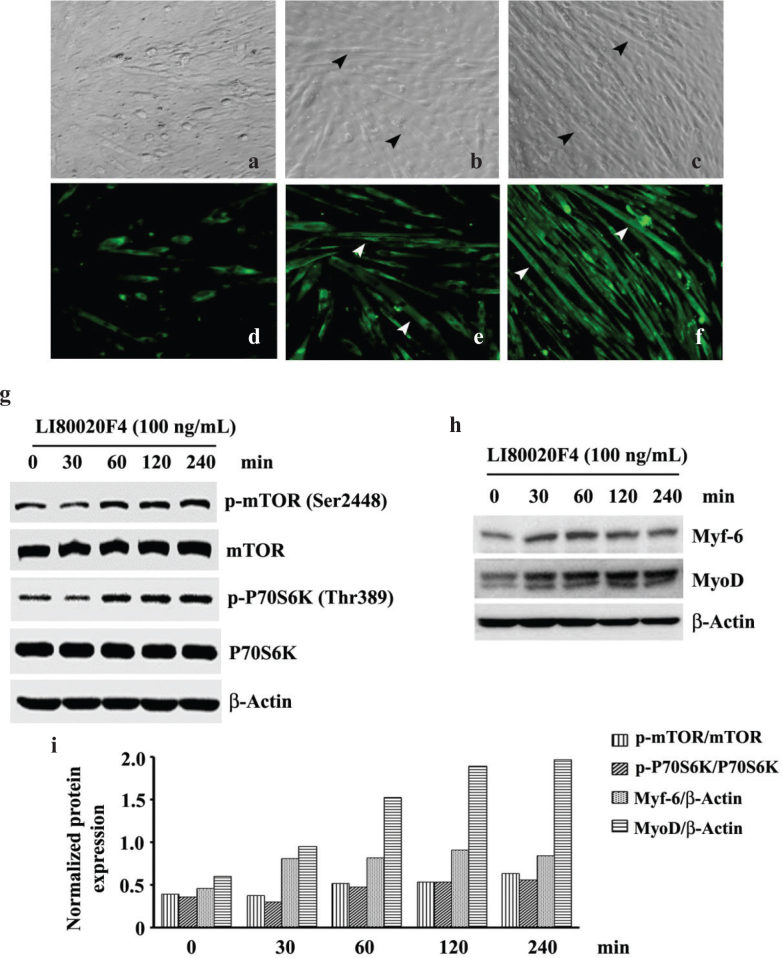
LI80020F4 induces C2C12 myogenesis, hyper-phosphorylates mTOR protein, and overexpresses myogenic factors. (a–c) show representative phase contrast photomicrographs; (d–f) show Myosin heavy chain (MYH) immunofluorescence staining of vehicle control (VC), 100 and 1,000 ng/ml LI80020F4-treated C2C12 myotube structures, respectively. The arrowheads show the multi-nucleated myofibers. Representative western blot images show time-dependent increases in phospho-mTOR (Ser2448) and phospho-P70S6K (Thr389) (g); Myf-6 and MyoD (h) protein expressions in LI80020F4-treated L6 skeletal myoblasts as indicated. The β-actin expression ensures equal cell lysate protein loading. (i) Bar graph presents normalized protein expressions in LI80020F4-treated cells at different time points, as indicated.

Mouse C2C12 cells are widely investigated as an *in vitro* model of myogenesis and skeletal muscle fiber formation (25, 26). Phase contrast (Fig. 2b and c) and myosin heavy chain (MYH) immunofluorescence (Fig. 2e and f) photomicrographs show increased myogenesis or myotube formations in 100 and 1,000 ng/ml LI80020F4-treated C2C12 cells, respectively. The images show LI80020F4-treated myotubes are longer, thicker, and increased in number, with brighter green fluorescence staining intensities indicating more expression of MYH than the VC cells. The arrowheads indicate the multi-nucleated myofibers. Figures 2a and d show the phase contrast and MYH-immunofluorescence images of the 0.2% DMSO-treated (VC) cells. Image analysis shows that 100 and 1000 ng/ml LI80020F4 treatment increased the myotube diameters by 34.8% (*P* = 0.0035) and 41% (*P* = 0.0002), respectively, as compared to the VC cultures (data not shown).

Representative immunoblot images show time-dependent increases in protein expressions of phosphorylated mTOR (Ser2448) and p70S6K (Thr389) (Fig. 2g). P70S6K is a downstream effector protein of mTOR. Expression of phosphorylated P70S6K affirms the activation of mTOR in the treated L6 rat skeletal myoblasts. Next, LI80020F4 treatment also upregulated time-dependent Myf-6 and MyoD protein expressions (Fig. 2h). These two myogenic factors are essential for muscle growth and differentiation (27, 28). The bar graph presents the normalized protein expressions (vs. total proteins or β-actin) in LI80020F4-treated cells at different time points, as indicated (Fig. 2i).

#### LI80020F4 induces mitochondrial biogenesis and increases mitochondrial function in L6 cells

**Fig. 3:**
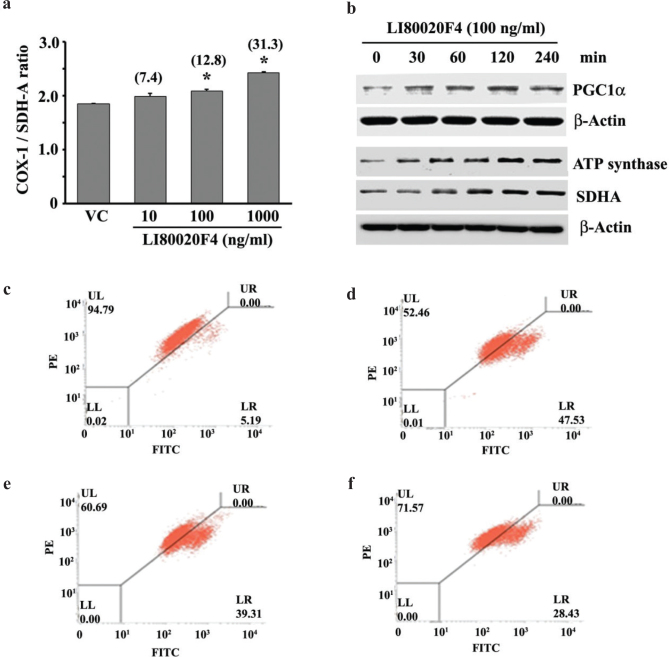
LI80020F4 enhances mitochondrial biogenesis and protects from oxidative stress-induced mitochondrial membrane depolarization in L6 cells. (a) Each bar presents COX-1/SDHA ratio as mitochondrial biogenesis (mean ± SD; *n* = 3) in the vehicle control (VC) and LI80020F4 (10, 100, and 1,000 ng/ml)-treated L6 cells. * indicates significance (*P* < 0.05, vs. VC) using paired t-test. Figures in parentheses present percent increase in mitochondrial biogenesis as compared to VC. COX-1, cytochrome c oxidase subunit I; SDHA, succinate dehydrogenase complex subunit A (b) Representative western blot images show increases in PGC1α, SDHA, and ATP synthase protein expressions in L6 cells, treated with LI80020F4 for different periods. β-Actin was used as the loading control. Flow cytometry dot-plots show JC-1-stained vehicle control (c) and 5 mM H_2_O_2_-treated L6 cells in the absence (d) or presence of 100 (e) and 1,000 (f) ng/ml of LI80020F4 for mitochondrial trans-membrane potential analysis.

Cells increase mitochondrial numbers by generating new mitochondria through the mitochondrial biogenesis process. Increased generation of new mitochondria is critical in enhanced energy production through the mitochondrial oxidative phosphorylation (OX-PHOS) system and energy utilization in cellular bioenergetics in rest and exercise (29). The mitochondrial biogenesis assay kit utilizes the principle of a ratio between mitochondrial DNA- and nuclear DNA-encoded proteins. The bar diagram presents mitochondrial biogenesis, represented by COX-1/SDHA ratio in the VC and LI80020F4-treated cells at different concentrations (Fig. 3a). The assay revealed that 10, 100, and 1,000 ng/ml of LI80020F4 treatments showed 7.4% (*P* = 0.0705), 12.8% (*P* = 0.0045), and 31.3% (*P* = 0.0001) increases (from VC), respectively, in mitochondrial biogenesis in L6 myoblast cells (Fig. 3a). The representative immunoblot images show upregulated PGC1α, ATP synthase, and SDHA protein expressions in the LI80020F4-treated cells in a time-dependent manner (Fig. 3b). PGC1α is a crucial regulator of mitochondrial biogenesis (29); ATP synthase and SDHA are the essential components of complex V and II of the mitochondrial OX-PHOS system (30).

Furthermore, to evaluate the protective efficacy of LI80020F4 in alleviating mitochondrial membrane depolarization, the L6 cells were treated with 5 mM H_2_O_2_ in the presence or absence of LI80020F4. Oxidative stress reduces mitochondrial membrane potential (∆Ψm) or depolarizes the membrane, decreasing JC-1 mitochondrial accumulation and J-aggregate formation, causing reduced red fluorescence (22). Figures 3c–f present the flow cytometry of JC-1-stained L6 cells. H_2_O_2_-treated cell culture showed a 47.53% depolarized population (Fig. 3d), contrasting with 5.19% depolarized cells in the VC (Fig. 3c). Whereas 100 and 1,000 ng/ml of LI80020F4-pretreated cultures in the presence of H_2_O_2_ contained 39.31 and 28.43% depolarized cell populations (Fig. 3e and f, respectively), indicating recoveries of 19.41 and 45.11% of cell populations, respectively, from the depolarizing effect of H_2_O_2_-induced oxidative stress.

### In vivo study

#### LI80020F4 supplementation improved muscle energy and strength

**Fig. 4:**
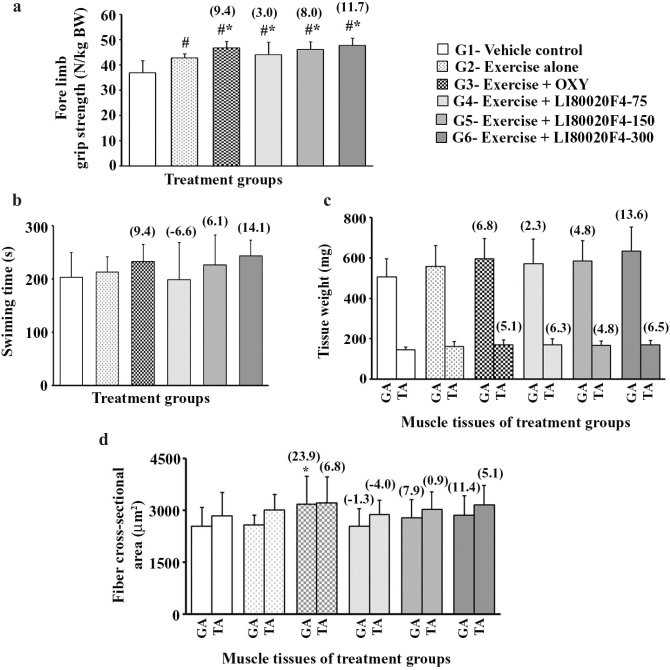
LI80020F4 supplementation increases forelimb grip strength, swimming time, and skeletal muscle mass in mice. The bar diagrams present mean ± SD of (a) forelimb grip strength (N/kg/bw), (b) swimming time (s), (c) gastrocnemius (GA) and tibialis anterior (TA) muscle weights (in mg), and (d) muscle fiber cross-sectional area (CSA, in µm^2^) of the experimental groups as indicated. Figures in parentheses present the percent change compared to the exercise control group. bw, body weight; OXY, Oxymetholone; *n* = 12; # and * indicate significance (*P* < 0.05) using the one-way ANOVA followed by the Dunnett post hoc test versus vehicle control (G1) and versus Exercise alone (G2) group, respectively.

Post supplementation, the fore-limb grip strength of the experimental mice was assessed as a measure of muscular strength, and the results are presented in Fig. 4a. The mean improvements in grip strength (N/kg bw) were increased by 3, 8, and 11.7% in the LI80020F4 75-, 150-, and 300 mg/kg bw groups, respectively, compared to the exercise control (G2) group. These increases are significant (*P* < 0.05) as compared to the VC and the exercise alone (G2) group mice. Similarly, the oxymetholone-supplemented mice also showed a 9.4% (*P* < 0.05 vs. G2) increase in muscle strength.

In the forced swim test (FST), the mid- and high-dose LI80020F4-supplemented groups increased 6.1 and 14.1% total swimming time, as compared to the G2 mice. Similarly, the OXY-supplemented animals showed a 9.4% increase (vs. G2) in swimming time. Although the 300 mg LI80020F4 group showed more sustained exercise, the gains are not significant compared to the exercise-alone group (Fig. 4b).

Post-supplementation, the GA and TA muscle weights of the supplemented mice were increased as compared with the G2 group (Fig. 4c). The bar graph presents 75-, 150-, and 300 mg/kg bw of LI80020F4 supplementation resulted in 2.3, 4.8, and 13.6% increases in GA muscle weights, 6.3, 4.8, and 6.5% increases in TA muscle weights, respectively, as compared to the exercise-alone group. OXY-supplemented animals also showed 6.8 and 5.1% increases (vs. G2) in GA and TA muscle weights. However, these muscle weight increases in the supplemented groups are not significant (vs. Exercise-alone group) (Fig. 4c).

Next, the morphometric evaluation reveals that 150 and 300 mg/kg of LI80020F4 supplementation induced 7.9 and 11.4% increases in GA muscle fiber CSA, 0.9 and 5.1% increases in TA muscle fiber CSA, respectively, as compared to the exercise-alone group. In the OXY group, GA and TA muscle fiber CSA were also increased (vs. Exercise-alone) by 23.9% (*P* < 0.05) and 6.8%, respectively (Fig. 4d). Except for GA muscles in the OXY group, the increases in muscle fiber CSA in the LI80020F4 and OXY groups are not significant versus the exercise-alone group.

#### LI80020F4 supplementation induced AKT/mTOR phosphorylation and modulated muscle growth-specific proteins expressions in gastrocnemius muscle

**Fig. 5:**
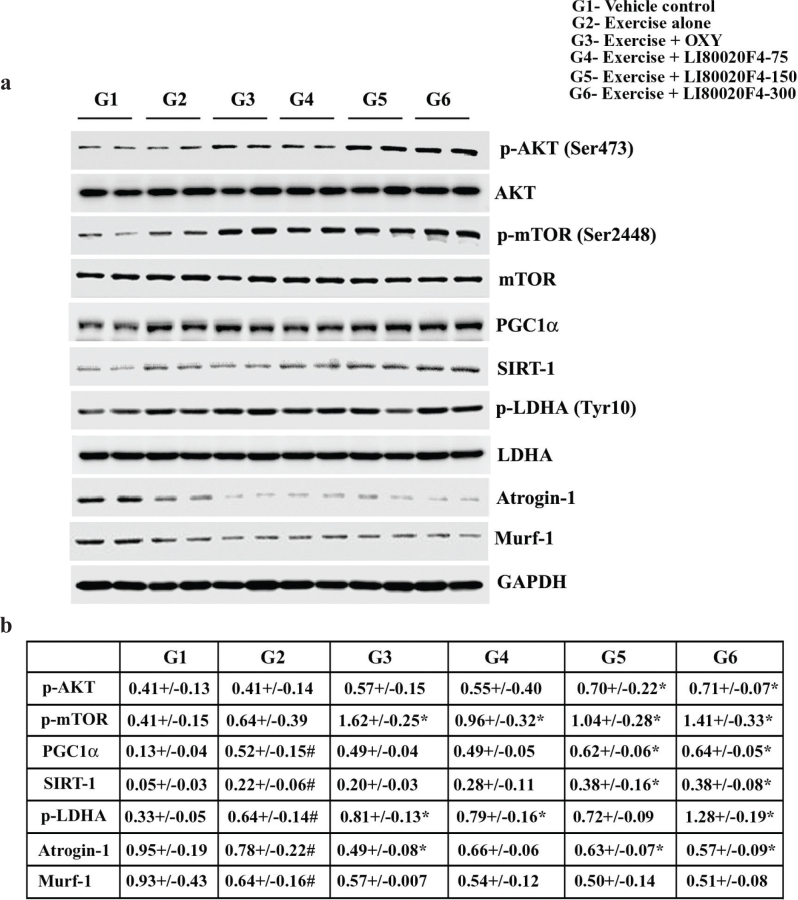
LI80020F4 supplementation induces AKT/mTOR phosphorylation and modulates muscle growth-specific protein expressions in gastrocnemius muscle. (a) Representative western blot images depict p-AKT (Ser473)/ AKT, p- mTOR (Ser2448)/ mTOR, PGC1α, SIRT-1, p-LDH (Tyr10)/LDH, Atrogin-1, and MURF-1 protein expressions in the gastrocnemius muscle lysates of the experimental mice as indicated. GAPDH protein expression ensures equal tissue lysate protein loading. The p-AKT (Ser473), p-mTOR (Ser2448), and p-LDH (Tyr10) were normalized with AKT, mTOR, and LDH protein expressions, and the remaining marker proteins were normalized with GAPDH. (b) Table presents the mean ± SD (*n* = 12) of the normalized protein expressions (in arbitrary units), group-wise, as indicated. # and * indicate significance (*P* < 0.05) using one-way ANOVA followed by the Dunnett post hoc test.

The representative immunoblot images show modulations of different protein expressions involved in muscle protein synthesis and degradation through anabolic and catabolic pathways. The images indicate that 150 and 300 mg/kg LI80020F4 supplementations increased protein expressions of phosphorylated AKT (Ser473), mTOR (Ser2448), PGC1α, SIRT-1, and decreased Atrogin-1 and Murf-1 protein expressions in the GA muscles (Fig. 5a). The table summarizes group-wise protein expression data (mean ± SD) and comparison analyses (vs. G2) showing differences in protein expressions in the supplemented animals. The data are derived from the densitometry analysis of the western-blot protein bands (Fig. 5b).

Furthermore, the immunoassays show that LI80020F4 supplementation modulated the levels of several markers in the GA muscle lysates (Table 1). Muscle glycogen levels increased by 23.08, 30.8, and 69.23% in the low, mid, and high dose groups (vs. G2). Also, the high-dose group showed a 15% increase in IGF-1 levels compared to the exercise-alone group. Also, in comparison with G2 animals, the low, mid, and high dose LI80020F4 showed 19.05, 26.19, and 47.62% (*P* < 0.05) reductions in lactate; 1.42, 8.19, and 21.97% reductions in Myostatin (GDF-8); and 15.86, 38.35 (*P* < 0.05), and 41.1% (*P* < 0.05) reductions in ROS levels; respectively (Table 1). Also, the GA muscles of 150 and 300 mg groups showed 44.73 and 43.26% reductions in MDA and 12.09 and 21.83% increases in catalase levels compared to the exercise-alone group; these changes are not significant (Table 1).

**Table 1:** Effect of LI80020F4 supplementation on gastrocnemius muscle tissue markers

Markers	Vehicle control	Exercise alone	Exercise plus			
			OXY-16	LI80020F4-75	LI80020F4-150	LI80020F4-300
Glycogen (µg/mg tissue)	0.07 ± 0.06	0.13 ± 0.09	0.14 ± 0.11	0.16 ± 0.08	0.17 ± 0.09	0.22 ± 0.08
Lactate (nM/mg tissue)	0.44 ± 0.12	0.42 ± 0.16	0.36 ± 0.18	0.34 ± 0.15	0.31 ± 0.16	0.22 ± 0.12*
Inorganic phosphorous (nM/mg tissue)	0.77 ± 0.22	0.95 ± 0.25	0.91 ± 0.26	1.27 ± 0.29*	1.29 ± 0.36*	1.18 ± 0.32
GDF-8 (pg/ml/mg protein)	53.34 ± 11.58	46.52 ± 13.33	44.67 ± 9.49	45.86 ± 8.89	42.71 ± 6.39	36.30 ± 6.28
IGF-1 (pg/mg protein)	176.5 ± 23.05	213.4 ± 51.77	234.6 ± 69.5	183.9 ± 47.91	202.8 ± 35.31	245.4 ± 90.29
ROS (µM/mg protein)	5.77 ± 2.43	6.18 ± 2.98	4.64 ± 2.09	5.20 ± 2.47	3.81 ± 0.94*	3.64 ± 1.74*
MDA (nM/mg protein)	7.61 ± 2.79	7.49 ± 3.46	7.66 ± 4.90	7.65 ± 5.96	4.14 ± 1.52	4.25 ± 1.33
Catalase (mU/mg protein)	3.23 ± 1.23	3.39 ± 2.20	4.07 ± 2.04	3.40 ± 1.52	3.80 ± 0.96	4.13 ± 1.65
Glutathione (µM/g protein)	27.41 ± 7.77	27.96 ± 6.43	26.96 ± 5.13	25.14 ± 5.31	28.75 ± 8.05	28.83 ± 7.02

The data is present as mean ± SD (*n* = 12). The treatment group descriptions are in the Materials and Methods section. GDF-8, Growth/differentiation factor 8; IGF-1, Insulin-like growth factor 1; ROS, Reactive oxygen species; MDA, Malondialdehyde. One-way ANOVA followed by Dunnett’s post hoc test;

* (*P* < 0.05) versus Exercise alone.

#### LI80020F4 improves serum biochemical markers

The 150 and 300 mg/kg LI80020F4-supplemented mice significantly decreased serum CK levels by 51.03% (*P* < 0.05) and 54.83% (*P* < 0.05) and LDH levels by 6.79 and 12.86%, respectively. In the LI80020F4-supplemented mice, serum AST, ALT, and BUN levels were also reduced in a dose-dependent manner. However, these changes were not significant as compared to the G2 group. LI80020F4 supplementation did not alter the inorganic phosphate levels (Table 2).

**Table 2:** Effect of LI80020F4 supplementation on serum markers

Measures	Vehicle Control	Exercise alone	Exercise plus			
			OXY-16	LI80020F4-75	LI80020F4-150	LI80020F4-300
CK (U/L)	164 ± 59.7	145 ± 67.5	121 ± 72.2	124 ± 82.5	71 ± 36.3^#^*	65.5 ± 36.4^#^*
LDH (U/L)	209 ± 42.4	280 ± 64.8^#^	273 ± 68.0*	253 ± 39.1	261 ± 64.3	244 ± 47.8
BUN (mg/dl)	27.67 ± 5.00	29.07 ± 3.71	25.33 ± 3.44	26.80 ± 4.62	25.73 ± 5.38	24.92 ± 2.90
AST (U/L)	98.07 ± 25.81	107.2 ± 27.8	105.4 ± 38.84	106.4 ± 43.34	103.2 ± 33.56	90.77 ± 19.66
ALT (U/L)	59.6 ± 25.77	68.8 ± 41.58	65.27 ± 40.98	69 ± 42.78	65.92 ± 35.3	60.4 ± 19.35
Pi (mg/dl)	8.96 ± 0.64	8.81 ± 1.07	8.89 ± 1.22	8.74 ± 0.87	8.53 ± 0.81	8.57 ± 1.02

The data is present as mean ± SD (*n* = 12). The treatment group descriptions are in the Materials and Methods section. CK, creatine kinase; LDH, lactate dehydrogenase; BUN, blood urea nitrogen; AST, aspartate aminotransferase; ALT, alanine transaminase; Pi, inorganic phosphate. One-way ANOVA followed by Dunnett’s post hoc test;

# (*P* < 0.05) versus Vehicle control;

* (*P* < 0.05) versus Exercise alone.

## Discussion

**Fig. 6:**
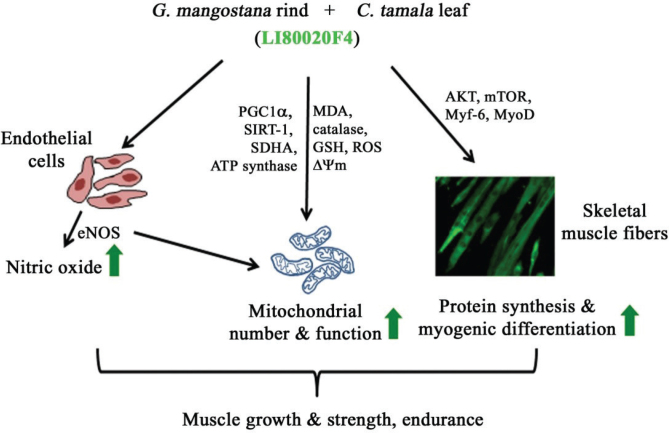
A schematic diagram represents the possible mechanisms of action of LI80020F4 in increasing muscle growth and strength, along with endurance. LI80020F4 1) boosts nitric oxide production in the endothelial cells via activating eNOS, 2) increases mitochondrial number, 3) mitochondrial function, 4) provides antioxidant defense and balances mitochondrial membrane potential, 5) increases myogenic differentiation via modulating key regulatory factors *in vitro* and *in vivo*. Green arrows indicate induction or increase. The abbreviations are described in the text.

The development of LI80020F4 and the earlier clinical study (16) were based on a postulation that a novel botanical composition that enhanced eNOS-dependent endogenous nitric oxide levels might improve mitochondrial function, muscle strength, and physical performance in exercising men. Earlier, in a proof-of-concept clinical study, this formulation increased muscle strength and endurance in males when supplemented with a 42-day resistance training program (16). The mechanism of action of LI80020F4 has not been explored. The present *in vitro* and *in vivo* observations explain the possible basis of the ergogenic potential of LI80020F4 (Fig. 6).

The present *in vitro* cell-based observations confirm that LI80020F4, a synergistic combination of *Garcinia mangostana* fruit rind and *Cinnamomum tamala* leaf extracts, enhances endogenous NO synthesis in endothelial cells. This observation is supported by eNOS activation in the LI80020F4-treated cells (8, 9). *G. mangostana* and *C. tamala* extracts are rich in polyphenolic constituents; this class of phytochemicals, including xanthones, is reported to exhibit various physiological activities that result in cardiovascular and antioxidant benefits (31, 32). In early investigations, α-mangostin, a major xanthone (33), and rutin (34) increased eNOS-dependent NO production.

One of the prime highlights of the present study is that LI80020F4 showed anabolic effects in both *in vitro* and *in vivo* models. Enhanced C2C12 myotube formation, increased expressions of the myogenic proteins *in vitro*, and mTOR activation *in vitro* and *in vivo* suggest a potential role of LI80020F4 in inducing myogenic differentiation. These data are supported by the observations that LI80020F4 increased muscle strength, muscle mass, and hypertrophied muscle fibers in the experimental mice. Our observations corroborate with an earlier study that demonstrated the role of NO in the myogenic differentiation of C2C12 myoblasts (35). The present *in vitro* and *in vivo* data suggest that LI80020F4 induces skeletal muscle hypertrophy in association with increasing mitochondrial function and upregulating mTOR signaling and myogenic differentiation factors through enhanced Akt activation and muscle IGF-1 levels elevation (36). In parallel with increased myogenic factor protein expressions, one interesting observation is that the LI80020F4 supplementation decreased both muscle Atrogin-1 and MURF-1 levels. Upregulated levels of these E3 ubiquitin ligases play a key role in protein degradation and skeletal muscle atrophy (37). An earlier study demonstrated that curcumin and gamma-oryzanol induced muscle hypertrophy via IGF1/Insulin-Akt-mTOR signaling and down-regulated Atrogin-1 and MuRF-1 genes in middle-aged rats (38). The present observations suggest a possible basis for skeletal muscle growth and strength in LI80020F4-supplemented mice and comply with the anabolic effect of LI80020F4 supplementation in the earlier reported clinical study (16). Moreover, the present data indicate a strong possibility of using LI80020F4 to prevent age-related loss of muscle mass and function.

Vascular endothelium-derived NO regulates mitochondrial biogenesis, mitochondrial respiration, and skeletal muscle function (39). During a sustained workout, mitochondria meet most energy demands through increased oxidative phosphorylation, essential for enhanced muscle adaptation and greater endurance (40). In the present study, enhanced mitochondrial biogenesis accompanied by PGC1α overexpression in L6 cells *in vitro* and increased phosphorylated AKT, SIRT1, and PGC1α protein expressions confirm a positive regulatory role of LI80020F4 in enhanced mitochondrial biogenesis in the skeletal muscles. In this context, it is of note that several natural extracts, including *Kaempferia parviflora* (41), *Rhodiola rosea* (42), and *Crocus Sativus* (43) extracts, have been reported to enhance mitochondrial biogenesis. LI80020F4 also increased protein expressions of SDH and ATP synthase in L6 cells, key enzymes of the complex II and V, respectively, of the mitochondrial electron transport chain. SDH is reported to be an indicator of mitochondrial mass (44) and has been linked with skeletal muscle growth associated with an increase in PGC1α (45). Besides, LI80020F4 treatment reduced H_2_O_2_-induced mitochondrial membrane depolarization in L6 cells, and in mouse GA muscles, the improved levels of oxidative stress markers indicate that this phytoceutical composition can protect muscle and mitochondrial function from exercise-induced oxidative stress. Reduced level of serum CK further supports significant protection from muscle damage; this is an important aspect of LI80020F4 in the supplemented animals. During intense workouts, excessive levels of free radicals generated due to mitochondrial respiration led to mitochondrial dysfunction, chromosomal damage, inflammation, and cell death (46). Finally, another critical aspect of LI80020F4 supplementation is that it normalized the serum levels of hepatic transaminases and BUN in the experimental mice, suggesting a protective efficacy of LI80020F4 on the muscular exercise-induced pathological liver (47) and kidney (48) functions.

## Conclusion

The present data provide scientific evidence that LI80020F4, a synergistic botanical composition, increases eNOS-dependent NO synthesis. The *in vitro* and *in vivo* data together show that LI80020F4 increases mitochondrial biogenesis, mitochondrial function, and myogenic differentiation and their regulatory protein expressions; the present observations demonstrate the underlying mechanism of actions and provide evidence that explains the basis of benefits of LI80020F4 supplementation in increasing muscle mass, strength, and endurance as reported in the earlier clinical study. Moreover, the present study shows that LI80020F4 provides antioxidant protection to the muscles mitigating exercise-induced muscle damage.

## Data Availability

Data and publication materials are available upon request.
